# Listening to Stakeholders Involved in Speech-Language Therapy for Children With Communication Disorders: Content Analysis of Apple App Store Reviews

**DOI:** 10.2196/28661

**Published:** 2022-01-21

**Authors:** Yao Du, Sarah Choe, Jennifer Vega, Yusa Liu, Adrienne Trujillo

**Affiliations:** 1 Monmouth University West Long Branch, NJ United States; 2 California State University, Fullerton Fullerton, CA United States; 3 Long Beach Unified School District Long Beach, CA United States; 4 San Francisco State University San Francisco, CA United States; 5 Newport Heights Elementary School Newport, CA United States

**Keywords:** eHealth, mobile health, mHealth, mobile app, communication disorders, speech therapy, language therapy, children, mobile phone

## Abstract

**Background:**

With the plethora of mobile apps available on the Apple App Store, more speech-language pathologists (SLPs) have adopted apps for speech-language therapy services, especially for pediatric clients. App Store reviews are publicly available data sources that can not only create avenues for communication between technology developers and consumers but also enable stakeholders such as parents and clinicians to share their opinions and view opinions about the app content and quality based on user experiences.

**Objective:**

This study examines the Apple App Store reviews from multiple key stakeholders (eg, parents, educators, and SLPs) to identify and understand user needs and challenges of using speech-language therapy apps (including augmentative and alternative communication [AAC] apps) for pediatric clients who receive speech-language therapy services.

**Methods:**

We selected 16 apps from a prior interview study with SLPs that covered multiple American Speech-Language-Hearing Association Big Nine competencies, including articulation, receptive and expressive language, fluency, voice, social communication, and communication modalities. Using an automatic Python (Python Software Foundation) crawler developed by our research team and a Really Simple Syndication feed generator provided by Apple, we extracted a total of 721 app reviews from 2009 to 2020. Using qualitative coding to identify emerging themes, we conducted a content analysis of 57.9% (418/721) reviews and synthesized user feedback related to app features and content, usability issues, recommendations for improvement, and multiple influential factors related to app design and use.

**Results:**

Our analyses revealed that key stakeholders such as family members, educators, and individuals with communication disorders have used App Store reviews as a platform to share their experiences with AAC and speech-language apps. User reviews for AAC apps were primarily written by parents who indicated that AAC apps consistently exhibited more usability issues owing to violations of design guidelines in areas of aesthetics, user errors, controls, and customization. Reviews for speech-language apps were primarily written by SLPs and educators who requested and recommended specific app features (eg, customization of visuals, recorded feedback within the app, and culturally diverse character roles) based on their experiences working with a diverse group of pediatric clients with a variety of communication disorders.

**Conclusions:**

To our knowledge, this is the first study to compile and analyze publicly available App Store reviews to identify areas for improvement within mobile apps for pediatric speech-language therapy apps from children with communication disorders and different stakeholders (eg, clinicians, parents, and educators). The findings contribute to the understanding of apps for children with communication disorders regarding content and features, app usability and accessibility issues, and influential factors that impact both AAC apps and speech-language apps for children with communication disorders who need speech therapy.

## Introduction

### Background

In recent decades, the Apple App Store has experienced a drastic increase in the number of mobile apps across multiple genres (eg, education, games, and health and fitness) for children. Many of these apps are designed for children with communication disorders; many are also used by their speech-language pathologists (SLPs) during assessments and interventions [1-3]. Different genres of mobile apps may be used for assistive, educational, and recreational purposes within the context of speech-language therapy depending on the communication abilities of children with communication disorders. For example, children with complex communication needs benefit from using augmentative and alternative communication (AAC) apps installed on mobile tablets (eg, iPad [Apple Inc]). Such devices enable AAC users to communicate with others via prestored symbols, pictures, and texts as an alternative communication modality [4,5]. In addition, educational speech therapy apps, including game apps that contain speech sound stimuli or language-based activities, have been implemented during therapy to target specific intervention domains [2,6,7].

The design and implementation of mobile apps for use by children with communication disorders is a research area that draws attention from both clinical researchers and human-computer interaction researchers. In recent years, human-computer interaction scholars have designed apps for children with autism, cleft palate, speech sound disorders, cochlear implants, and other communication disorders [8-12]. Given the variety of needs among children with communication disorders, developers and designers may encounter difficulties obtaining verbal or written user feedback on app content and features while creating and revising these apps; consequently, they must rely on reports from key stakeholders that surround the circle of care of children with communication disorders [13,14]. Some stakeholders included within the circle of care of children with communication disorders are SLPs; parents; teachers; and, sometimes, the children with communication disorders themselves. Involving all stakeholders in the initial design process would be costly, time-consuming, and unwieldy, and there are multiple obstacles to conducting empirical user studies examining the app use experience of children with communication disorders directly [15-17].

App Store reviews offer an opportunity to investigate app user experience from a multi-stakeholder perspective, which has heretofore been unexamined. App Store reviews are publicly available data sources from customers, serving as a communication avenue for app users to express their needs and challenges with the apps they purchased and downloaded. App Store reviews not only influence decisions by other users regarding app purchases but also bring awareness to developers about critical issues related to app design and development, including but not limited to criticism of current app features and functions and ideas for new app features.

### Relevant Work

Previous user review studies have examined thousands of app reviews from different genres of apps on the Apple App Store [18-20] and the Google Play Store [18,21-23]. Popular apps can receive a large volume of reviews daily. However, analyzing these linguistic data and categorizing reviews in large amounts may be difficult, specifically when dealing with varying quality of reviews and with mixed sentiments within a single review [18]. Researchers have used manual coding as well as automated data mining techniques (eg, natural language processing for topic, semantic, and sentiment analysis) to analyze linguistic data on a large scale, categorize various user intent, and organize user feedback for feature extraction [21,22,24,25]. Studies that analyze popular game, social, communication, and productivity apps (eg, Angry Birds [Rovio Entertainment], Facebook [Meta Platforms], Pinterest, WhatsApp [Meta Platforms], and Dropbox) have suggested that user reviews offer valuable feedback for information giving, information seeking, feature requests, and problem discovery, along with rich contextual descriptions of feature requests and ideas for improvements [18,20,22]. Fu et al [21] further found that even user complaints can be useful, as the number of complaints were highest following a release, with top complaints primarily related to content attractiveness, app stability, and cost. Khalid et al [20] found similar trends, with more than half of the complaints addressing functional errors, feature requests, and instances of the app crashing.

Although previous research on app reviews has focused on non–health-related apps, research is lacking regarding user reviews for apps targeting individuals with disabilities or apps related to health-related interventions. One area of research that is growing is regarding the efficacy of mental health apps. For example, researchers have begun to investigate the potential positive impact of mental health apps, particularly in increasing access to mental health interventions. Although prior studies on user reviews for general popular apps can be useful to guide review analysis for health-related apps [19-22,26], research on apps in health intervention highlights the additional importance of user engagement, especially among consumers who have specific health needs or disabilities [27,28]. Torous et al [29] and Stawarz et al [23] examined the effectiveness and user engagement of cognitive behavioral therapy apps and found that in addition to poor usability and failure to meet user needs, users also have low engagement and rising uncertainties about the effectiveness of mental health apps [29]. These studies have identified the need for further exploration of how App Store reviews might increase designer and developer knowledge of user issues, enabling evidence-based design practices that could increase the user-reported efficacy of health-intervention apps.

Despite the vast number of commercial mobile apps available for children, there have been very few published studies related to user-reported satisfaction regarding the efficacy of speech-language therapy apps for children. A recent study [30] examined the content and quality of mobile apps for speech-language therapy in adults with communication disorders; however, to our knowledge, no published studies have examined publicly available user reviews to understand the user experience with speech-language apps of children with communication disorders. Investigating the review content for pediatric speech-language apps not only allows adult stakeholders to share the user challenges of children with communication disorders with app designers but also enables researchers to understand the communication needs of both children and adults. Children with communication disorders typically depend on their caregivers, educators, and therapists to make decisions regarding app recommendations; however, owing to the lack of up-to-date research regarding systematic guidelines for app selection and evaluation, clinical decision-making can be difficult for the adult stakeholders of children with communication disorders [31]. Parents often make purchase decisions based on usability reviews and ratings from the App Store, and even clinicians have to rely on word of mouth, marketing offers, or cost to make decisions when purchasing apps for use during therapy [1].

Investigating user reviews across different genres of mobile apps can inform design practitioners about specific usability issues that may impede the interaction of children with communication disorders with the apps, and helps clinicians learn about app content and features to make clinical recommendations that best serve their clients’ needs. Previous studies have reported that app users often use the same linguistic patterns to communicate a problem but that linguistic patterns may vary more when making feature requests. This variance makes automatic analysis difficult to successfully identify and categorize user perspectives on feature requests [18,22]. This study uses automatic review extraction and manual review screening and analysis to examine user reviews from a selected set of mobile apps for pediatric speech therapy from the Apple App Store. By identifying app feature requests and critical usability issues, as well as multiple influential factors (eg, financial, sociocultural, ethical, and political) affecting user experiences, this study seeks to inform designers and developers who aim to create child-centered and clinically informed speech-language apps for children with communication disorders.

## Methods

### App Selection and Screening

**Figure 1:**
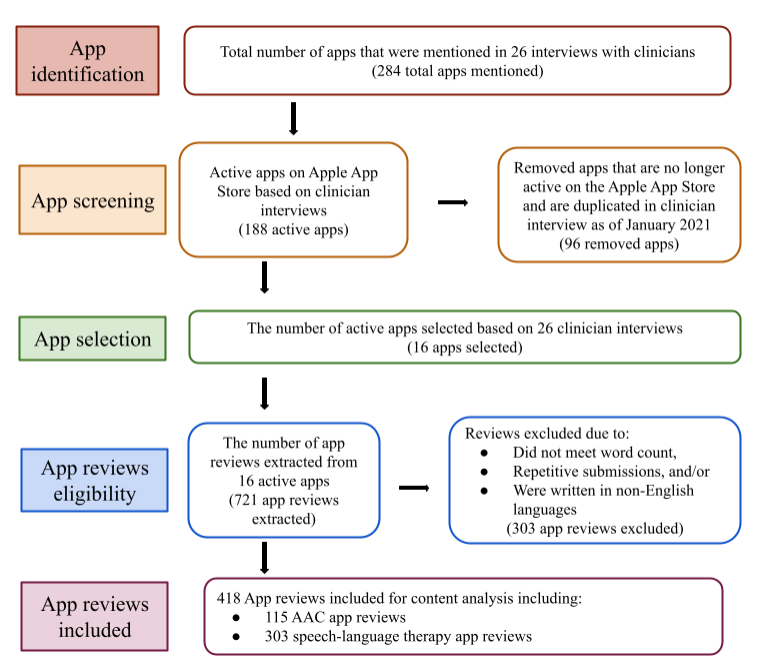
A flowchart for app selection, app review extraction, and content analysis. AAC: augmentative and alternative communication.

This study builds on a prior qualitative interview study with 26 SLPs who reported a total of 284 mobile apps they use with children during speech and language therapy [15,16]. These participating SLPs ranged across multiple settings such as schools, private practices, hospitals, and home health services from various states in the United States (Multimedia Appendix 1). Using apps collected from the SLPs’ interviews enabled us to examine technological tools that clinicians reported using rather than querying app searches through researcher-designed keywords on the Apple App Store. We used multiple verification and categorization steps for app selection and screening, as indicated in Figure 1. First, we verified whether the 284 apps mentioned by SLPs were inactive or active on the Apple App Store. Inactive apps were apps that were no longer available on the Apple App Store, whereas active apps were apps available for consumers to download and use as of January 2021. We identified and excluded 33.8% (96/284) apps that were no longer active and classified the remaining 66.2% (188/284) active apps into four categories as follows: (1) AAC apps, (2) speech-language apps, (3) game apps that do not contain therapy content, and (4) utility apps. These four categories are consistent with prior research on app reviews for individuals with visual impairments, as Torres-Carazo et al [27] have reported that these individuals use games and utility apps in addition to various kinds of assistive technology apps. A previous study [1] suggested that in their university clinic, AAC apps (eg, Proloquo2Go) were the apps most frequently checked out by speech-language pathology clinicians. This study stated that student clinicians also preferred speech-language apps with content-specific visual feedback and apps that allowed them to target a variety of speech and language therapy goals [1]; therefore, researchers must consider both AAC apps and speech-language apps, as both genres of apps are designed for speech-language intervention and are frequently used by SLPs when working with children with communication disorders.

Next, to further categorize these apps into specific speech and language therapy domains, we followed the Big Nine intervention domains from the American Speech-Language-Hearing Association [32]. The Big Nine domains include articulation, fluency, voice and resonance, receptive and expressive language, hearing, swallowing, cognitive aspects of communication, social aspects of communication, and communication modalities [32]. The final 5.6% (16/284) of selected apps had the most user reviews and covered multiple American Speech-Language-Hearing Association Big Nine domains in the areas of articulation, receptive and expressive language, social aspects of communication language, and communication modalities. These 16 chosen apps include 7 (44%) AAC apps (Multimedia Appendix 2) and 9 (56%) speech-language therapy apps (Multimedia Appendix 3).

### App Review Extraction

To ensure that the 16 selected apps had adequate app reviews, we organized these apps from the most to the least number of web-based app reviews available for content analysis. We used a Really Simple Syndication feed generator provided by Apple and an automatic Python crawler developed by our research team, which enabled app reviews and other related information, such as review date, reviewer name, review title, and content, to be extracted and exported to the comma-separated values file format for the iOS apps selected in this study. We extracted a total of 721 app reviews from all 16 apps, with review dates ranging from 2009 to 2020.

After app review extraction, we first manually reviewed and excluded 42.2% (304/721) of the app reviews, including 8.3% (60/721) AAC app reviews and 33.8% (244/721) speech therapy app reviews. These reviews were excluded because they were too short, repetitive, written in languages other than English, or not applicable for pediatric speech therapy. We then conducted a content analysis of a total of 418 reviews, including 115 (27.5%) AAC reviews and 303 (72.5%) speech therapy app reviews, all with at least 20 words per review to ensure adequate content was included in each review (Figure 1). For the app *Articulation Station* and *Word Vault Essential*, only reviews from the free versions were included because of an insufficient number of reviews for the pro version compared with the free version (Multimedia Appendices 2 and 3). This is consistent with a prior research [19], which discovered that more reviews were written for free apps than for paid apps; as a result, apps in the games category, which are typically free, tend to receive the highest number of reviews compared with other app categories where apps more frequently must be purchased.

### Qualitative Coding of App Reviews

This study used manual coding for app reviews; for manual coding to be effective, it is vital to have a coding system that provides clear definitions and examples when determining what should be classified under each code. Although discrepancies can occur during manual analysis, a coding guide is one way to help limit the number of times reviews will need to be looked at by multiple individuals. The research team consisted of one licensed SLP, YD, one graduate clinician, AT, and three remaining authors, SC, JV, and YL, who were licensed SLP assistants. The qualitative coding process involves several steps. First, we used a subset of apps, including 19% (3/26) AAC apps (*Proloquo2Go*, *GoTalk* NOW, and *Language Acquisition Motor Planning Words for Life* [LAMP WFL]) and 19% (3/26) speech-language apps (*Articulation Station*, *Language Empires*, and *Between the Lines Level 1 HD*), the SLP and the graduate clinician generated initial codes using a deductive coding scheme from a prior interview study [16,33] and literature in universal design guidelines [33,34] and educational app evaluation heuristics [35]. After the initial codes were developed, the 3 SLP assistants coded the remaining reviews of the other apps and used the constant comparison method [36-38] to merge codes into broader categories and developed a codebook (Multimedia Appendix 4). Disagreements with content analysis were resolved using the negotiated agreement approach [39]. Individual codes in the codebook covered several areas including client characteristics (eg, age, type of disability, level of ability, and length of app use), clinician characteristics (eg, clinical setting, clinician location, clinician specialty, and length of app use), additional stakeholders (technical vs nontechnical personnel), clinical practice (eg, intervention area and domain, therapy goals and activities, workplace productivity, evidence-based practice, and research), app characteristics (eg, genre, content, use technique, and data management), device issues (eg, hardware vs software), usability issues (with a focus on control, error, aesthetics, customization, and accessibility), and recommendations (eg, app referral, suggestions for improvement, and feature requests). In addition, each app review was labeled when the review content specified the identity of an individual reviewer (eg, an SLP or a parent). In the next section, we discuss specific findings related to app content and features, usability and accessibility issues, and recommendations for future improvement mentioned by the users based on 2 genres of AAC and speech-language apps. Finally, we concluded by discussing multiple influential factors (eg, financial, sociocultural, ethical, and moral) that affect the implementation and use of both AAC apps and speech-language apps.

## Results

### App Characteristics: Content and Features, Usability, and Recommendations for AAC Apps

#### AAC App Content and Features

In this study, app reviews were coded based on information related to the app genre, app use technique, app content, and data management. For AAC apps, reviews were written by a range of critical stakeholders for children with communication disorders, including parents, SLPs, and AAC specialists, as well as AAC app users themselves. Identification of stakeholders was determined by the AAC reviews’ content (eg, pronouns mentioned, explicitly self-identified role, or unique characteristics). A total of 112 stakeholders were identified in the AAC reviews. The number of app reviewers were classified into six specific categories. Of the 112 stakeholders, the top 3 stakeholder groups who reviewed AAC apps included 51 (45.5%) parents, 24 (21.4%) AAC users, and 18 (16.1%) SLPs. In contrast, of the 112 stakeholders, only 1 (0.9%) educator, 2 (1.8%) AAC specialists, and 16 (14.3%) unknown stakeholders left app reviews. App reviewers were marked as unknown when there was insufficient information to identify which stakeholder group they were a part of.

Users of 56% (9/16) of AAC apps cover a wide age range, from toddlers to school-aged children and young adults. Users’ characteristics include various different types of physical and communication-related disabilities (eg, autism, language delay, Down syndrome, cognitive impairments, and mutism). When reviewing the 9 AAC apps, app reviewers compared design features and app characteristics across AAC apps (eg, *Proloquo2Go*, *Speak For Yourself*, and *TouchChat HD*). For instance, reviewers praised the app *Proloquo2Go* for its popularity in educational settings across elementary, junior, and high schools. A parent of a 6-year-old nonverbal autistic son praised the *Proloquo2Go* app, saying that it brings “the power of communication and made it accessible to some of the most vulnerable people in society.” Specifically, reviewers stated that AAC apps such as *Proloquo2Go* provide users the opportunity to use a list of core vocabulary words or high-frequency words (eg, want and more) in an “easy to add or rearrange” layout. Similarly, 10 reviews reported positive features, such as easy customization and personalization in the app LAMP *WFL.* Some individuals use the LAMP *WFL* app as a primary source of communication, whereas others use it to supplement other modes of primary communication modalities, such as sign languages. Unlike *Proloquo2Go*, LAMP *WFL* has a fixed display with static core words to support the user’s motor planning. This facilitates an evidence-based approach for AAC intervention, which was reported to be beneficial by parents. One parent specifically stated the following:

I like that the buttons on the original page can't be changed/moved around like in other apps. It helps the child memorize the placement of the buttons, so less frustration.

#### AAC App Usability Issues

Despite these positive reviews, user complaints were also reported, including usability issues such as missing content (eg, lack of fringe vocabulary or low frequency words) or unsatisfying content in relation to the high cost of AAC apps. App reviewers also noted difficulties accessing AAC apps across different types of devices owing to a lack of compatibility. Consequently, we categorized these hardware issues under the Level 2 code device, which primarily looks at AAC app issues related to hardware and software. An example of how this affects AAC users is that if they need to transition to a new device (eg, after breaking their current device), previously programmed app content may be lost or abandoned. To address this concern, apps such as *CoughDrop* and *GoTalk NOW* offer cloud-based storage systems to connect to Wi-Fi automatically. During the COVID-19 pandemic, many clinicians who have used these speech-language therapy apps with their clients in settings such as schools and private practice clinics have transitioned into providing therapy services on the web. One SLP who specializes in AAC and assistive technology used *CoughDrop* via teletherapy and stated the following:

Through telepractice ST services, I work with students across state lines and this app allows me the ability to create and synchronize boards from the comfort of my home to students in other states. This past year after collecting data in the reports sent by the app, two of the districts I contracted with purchased life-time apps for three students on trial. So what makes it a Guru AAC App? It is cloud based, user friendly, one can use the picture library within the app, or use your own cam pictures or web pictures, not to mention the great 1:1 communication from its developers any time I have reached out to them.

Enabling cloud-based data management solutions for AAC apps reduces the burden of transferring AAC data for both users and their therapists, which is especially beneficial during teletherapy.

AAC app reviews highlighted usability issues primarily in critical areas of user errors, controls, aesthetics, customization, and accessibility (Table 1). A total of 69 AAC reviews were coded for usability issues. The most common user errors for AAC apps were related to issues such as navigation and controls (eg, scrolling), selection (eg, icons), and delays during app use (eg, importing photos). These errors led to increased user frustration that impacted the ability of children with communication disorders to engage in functional communication. Analysis of 56% (9/16) of AAC app reviews related to user errors highlighted the importance of improving vocabulary and message selection and increasing efficient navigation and control to offer timely communication experiences for users with disabilities. Some recommendations in the reviews included allowing users to choose photos and edit icons to add vocabulary. Other challenges related to user control were reported to affect users’ ability to personalize and customize AAC apps. Some complaints and recommendations included updates that changed button placement that were previously learned, inability to increase low volume in noisy environments, fast pace of programmed speech rate, and lack of personalization and inclusion of user-specific voice output for aesthetic purposes. App reviews for *Speak For Yourself* and *TouchChat HD* emphasized the lack of diversity in options from the voice bank that failed to make users feel represented via their own voices when using AAC systems. For example, one review left on *TouchChat HD* requested voices that could represent children rather than adults, and another reviewer for Proloquo2Go mentioned the following:

More subtle adjustment of pitch is required - I don't want to sound like a mickey mouse at the level above normal! The acapella female voices do need to improve.

These reviews reflected user needs for controlling and using high-quality synthesized speech to represent voice profiles for users across different age groups and genders.

In addition to AAC app users, other stakeholders have also experienced app malfunction when using the app, which interfered with their clinical practices while trying to gather accurate data. Clinicians have reported varying user challenges, such as the inability to control language and images that were considered inappropriate for certain individuals. App reviews also revealed differences in perspectives between SLPs and parents. For instance, the app *LAMP WFL* has 15 reviews from parents reporting dissatisfaction, such as AAC item images that they consider inappropriate for their children; clinicians and special education teachers reported more dissatisfaction regarding therapy target areas that could be addressed via this app and recommended more social content to be included. In addition, caregivers specifically emphasized the critical need to have the support of a knowledgeable clinician to successfully program the AAC system onto its designated device. A user who downloaded *CoughDrop* felt that programing this particular AAC app can be challenging “unless you have a therapist that’s well versed in making changes.”

**Table 1:** User reviews on usability issues and recommendations for augmentative and alternative communication (AAC) apps.

AAC app name	App usability		App recommendations	
	Characteristic	Review	Characteristic	Review
CoughDrop	Compatibility	“Half of the time the changes I make on either the computer or iPad do not stay changed. With it only doing a few features it should be a lot easier to navigate.”	Programing	“Keep looking unless you have a therapist that’s well versed in making changes I wouldn’t bother with this.”
TouchChat HD	User errors	“The only problem is that when I edit a button in one screen my changes affect absolutely unrelated button in another screen, which is obviously a bug.”	Compatibility	“In the very least, if you purchased the $300 iPad app, they should offer a credit for the same iPhone app.”
Speak for yourself	Visual aesthetics	“Too many buttons. They don’t make sense (e.g., ‘little’/mouse picture goes to page with about a hundred flowers???? What is that about?!!!!!).”	Aesthetics	“If I could change three things, it would be the voices, the rigidity, and the grammar.”
Tobii Dynavox compass connect	Efficiency	“It's been an hour since the app finished downloading and I STILL haven't been able to get started on it.” “I downloaded it on my new iPad and when I try to open it, the wheel just spins and then it closes down.”	Data storage	“Not [recommend] takes up [too] much storage takes forever to install.”
LAMP WFL^a^	Customization	“However, the main drawback is that for a 12 -year -old, the app's vocabulary is severely lacking. There are not enough words. (For example the words blueberry and coyote are not in the vocabulary along with other words.)”	Volume	“My son loves this app but the volume is kinda low. Today I put it on maximum volume but could barely hear him while driving. It’s fine if your at home with no noise otherwise it’s difficult to hear. Please fix.”
Proloquo2Go	Navigation and control	“The sensitivity on this app is extremely annoying. Just scrolling will cause a button to be pushed at random. It’s quite annoying when you’re trying to use this and random buttons are getting pushed not by you intentionally.”	User errors	“My one problem is that it’s too easy for her to get into edit mode and start deleting/editing or adding nonsense buttons. Could you please add a password option to get into edit mode?”
GoTalk NOW	Programing	“Go Talk Now is an easily programmable communication app for sure. Updating buttons is quick and there are video guides to show how-to’s of creating books, even a free lite version to trial it.”	Data storage	“On my wish list for GTN is an easier way to backup communication books of more than 5 pages at a time for an iPad 2.”

^a^LAMP WFL: Language Acquisition Motor Planning Words for Life.

### App Characteristics: Content and Features, Usability, and Recommendations for Speech-Language Apps

#### Overview

In contrast to AAC apps, a total of 188 App Store reviews for speech-language apps were written by 51 (27.1%) SLPs, 39 (20.7%) educators, 32 (17%) parents, 5 (2.7%) other professionals, and 61 (32.4%) unknown stakeholders. This distribution of stakeholders is likely because, in addition to parents who actively seek apps to help their children, SLPs are the primary users of these apps. In the reviews for speech-language apps, clinicians not only recommended app content and features that addressed their clients’ needs but also provided app critiques so that the app designers and developers could improve apps to align with clinical practice guidelines for speech and language intervention. In the following section, we describe reviewer feedback related to content and features and usability issues in areas of user errors, aesthetics design, and desirable control and customization.

#### Articulation Apps

Articulation apps were reported to be used by children from ages 2 to 6 years, including individuals who, according to the reviews, have autism, speech delays, articulation disorders, and apraxia. The desirable content and features of these articulation apps focused on four key components: (1) include a variety of articulation targets speech sounds and corresponding letters, along with a plethora of target words; (2) offer consistent verbal models and opportunities for high volumes of repetition; (3) enable the selection of speech sound targets based on the ability level of a client (eg, sounds in isolation, in all word positions, and at the sentence level); and (4) allow clients to progress at multiple linguistic hierarchy levels by following the development of phoneme acquisition and evidence-based practice guidelines. For example, clinicians especially enjoyed the function of recording voice production and data tracking in the app Articulation Station, as it offers direct feedback for children with communication disorders to improve perception of their own speech production. One SLP stated in the review that “the students love the record function and it is useful for them to hear back their own productions.” Clinicians also commented that having the data collection and tracking functions help them save time and allow them to compare data across multiple sessions to measure overall therapy progress. In addition, reviewers commented that picture-card apps such as *Word Vault Essential* are convenient and portable for use with various clients for multiple therapy goals beyond articulation and phonology, as some of the word lists can be used to target language and pragmatic intervention. In addition to SLPs, educators (eg, teachers and reading specialists) and parents also left reviews for these speech therapy apps, and parents especially praised that these articulation apps could be incorporated multiple times daily outside speech therapy sessions at school and, therefore, become a supplement to help complete speech therapy exercises at home.

In terms of usability, there were 11 reviews that praised the aesthetic design of apps, such as the *Lively Letters-Phonics* app and stated that the app content was stored in a “neat, organized fashion” and that the in-app activities were “visually appealing, clear, and fun;” 2 recent reviews from July 2020 specifically mentioned that the app *Lively Letters-Phonics* was “a huge help during remote learning with the ability to use the lively letters on screen” in the pandemic context it was “so easy to use with Zoom meetings. The students were engaged and the activities helped to keep the lesson moving.” A total of 21 reviewers, mostly SLP clinicians, raised concerns regarding the app *Speech Blubs: Language Therapy* and its poor animation, incorrect pronunciations, inconsistent and low volume of sounds, and unnatural quality in voice recordings when computer-generated artificial speech is used instead of human voice recordings. One SLP raised a specific concern about the *Speech Blubs: Language Therapy* app by questioning the evidence-based design within the app, as well as the validity of parent reviews:

I am a speech-language pathologist. I was quite interested in this app to use in sessions and for parents to use at home. After looking through it, I wouldn't recommend it and am surprised other therapists do recommend it. First off, it really does not follow developmental speech milestones. It seems random to me and is not consistent with anything I do with children who are diagnosed with delays of expressive language, receptive language, apraxia, autism etc. Furthermore, from most of the reviews I read, it seems most parents are using this for their toddlers to develop language and first words. This app does not follow the typical developmental milestones of speech. It should start with early developing sounds to the early developing consonant-vowel combinations mixed with age appropriate play and functional communication words. I am sure that initially, children will say a few new words or sounds just from the novelty of the app (which occurs in speech therapy as well). However, I would like to hear from these parents after a month or so to see if progress continues or if they see some initial progress, wrote a positive review, then progress plateaus and they just cancel the subscription and forget about it. Because here’s the thing, I feel like the majority of what I do with young children who aren’t talking, is teaching parents how to play with their children with real toys, teaching how to securely attach (bond) with your child through play (no screens!), and how to elicit language during play. Children already get too much screen time and need to be interacting with and playing with real people and real toys. We need to be following developmental milestones with real play and interactions to build language and attachment together because communication is purely social so it needs to be done socially- not on their own with an app. This app will lead to mimicry but likely not lead to functional communication. This is my professional opinion. It appears other therapists find some merit in it (were they compensated for their reviews?) but I do not see how this could lead to functional communication and do not feel it should be called a speech therapy app.

Reviewers also complained about the app *Word Vault Essential* and reported incidents of erased data, frequent errors, too many advertisements, and incomplete content information, all of which affected user experiences for both clients and clinicians. Across all articulation apps, reviewers identified four usability issues and recommended the following user control and customization features: (1) enable selection of speech sound targets based on the client’s ability level (eg, sounds in isolation, in all word positions, and at the sentence level); (2) allow pause and resume content to meet the clients’ own pace; (3) hide certain visual stimuli (eg, junk food, rifles, and mythological characters) from younger children; and (4) integrate the use of games and reward systems to increase client engagement. These issues related to control, error, and customization were present in 21 reviews.

#### Receptive, Expressive, and Social Language Apps

Receptive, expressive, and social language apps were reported to be used by different age groups of users, including students in kindergarten, elementary, middle, and high school with autism, Asperger, and communication disorders spanning varying domains (eg, articulation, fluency, language, and social communication). For example, 1 SLP reported using the app *ChatterPix Kids* with students who have cochlear implants or hearing aids to encourage oral communication, although this app is not specifically designed for children with hearing impairments. Another SLP wrote a review for *ConversationBuilder* stating the following about the app:

A wonderful tool to use with my ASD students...what an amazing tool it is for facilitating other speech needs in a more spontaneous and naturalistic way: especially for my students with fluency issues...Also amazing for carryover of articulation and other expressive language needs.

Reviews of these language apps came from multiple adult stakeholders such as SLPs, educators, and parents, and they commented that apps such as *Language Empires*, *ConversationBuilder*, and *ChatterPix Kids* were easy to use by professional educators and clinicians as well as by caregivers (eg, parents). As versatile as articulation apps, these language apps can be used with clients with mixed language ability levels via individual or group therapy in schools, clinics, and at home. Specifically, these apps were used to target various language goals (eg, vocabulary development, semantic relationship, sentence production, story retell, conversation expansion, and turn-taking), as well as social pragmatic skills (eg, listening to intonations, inferencing figurative speech, reading body language in social scenarios, and sequencing social stories). Reviewers also shared that apps such as *ChatterPix Kids* are customizable with individualized pictures and voices, allowing endless possibilities for creative play (eg, making a skit with multiple people and making videos to express feelings) in addition to promoting communication via listening and speaking.

Language app reviewers described the following desirable content and features: (1) ability to integrate web-based data collection; (2) ability to record and replay answers for users to identify correct and incorrect responses; (3) ability to allow users to choose the level of difficulty in clinical practice; (4) ability to allow users to import and upload their own photos and videos to differentiate between fiction and nonfiction concepts; (5) ability to allow users to save and email conversations generated through app use to reinforce skill use from home to school; (6) ability to provide more concrete differentiations for answer choices; and (7) ability to use reward systems (eg, trophies) as motivators to attend to and complete tasks. In reviews for the *Social Detective* app, one reviewer commented that the app “follows the concepts in from the book and asks the user to make a smart guess using their social tool boxes about different social behaviors and interactions” suggesting this app has been used in conjunction with a physical companion workbook based on the Social Thinking curriculum to reinforce student learning. However, owing to limited content, 4 reviews consisted of requests for more up-to-date content in the *Social Detective* app’s video modules and specifically highlighted the need to diversify characters with more people of color in video modules to take on various character roles (eg, engaging in expected and unexpected behaviors). In addition to such features, users also requested automated reading features, despite the fact that many of these apps were designed to be used by children with adult assistance. In a review of the *ConversationBuilder* app, one English-as-a-second-language teacher in the elementary school commented the following:

One thing that would improve this app is if the language choices were read aloud by the iPad so that non-readers could access the app independent of a peer or adult who can do the reading.

Another parent also commented that she hopes that her 4-year-old daughter would be able to:

Touch the screen and hear the sentences. I am fine reading to my daughter but would like her to try reading on her own and getting hints if she needs them.

In terms of usability issues across 3 language apps (*Between the Lines, Level 1 HD*, and *ChatterPix Kids*), user *errors* were commonly seen with reports of the inability to save, email, and print the data of incidents of accidental removal or deletion of the data by the app. Clinicians suggested the following control and customization features across all the language apps: (1) customize controls to turn on or off background scenes to minimize visually distracting illustrations; (2) adjust sound effects for correct versus incorrect responses; (3) allow adequate response time for users; and (4) remove inappropriate slang and random reinforcers. It is worth mentioning that the adult stakeholder group had mixed reviews for the app *ChatterPix Kids*. Although one reviewer (identity unknown) stated that the app is “a perfect way to let the kids create fun stories in a safe and child friendly environment,” another teacher warned parents that the app is not appropriate for children as user-generated video contents can easily contain profanity. Although not directly impacting usability, these mixed reviews highlighted additional user needs around content moderation and monitoring in these speech-language apps (Table 2).

**Table 2:** Feature requests and recommendation from speech-language app reviews.

Intervention domain	Feature requests and recommendation	Sample quotes from reviewers
Articulation apps (Articulation Station, Lively Letters-Phonics, Speech Blubs: Language Therapy; and Word Vault Essential)	Add letters and a plethora of target words Offer consistent verbal models and a high-volume repetition Allow voice recordings for self-monitoring of own production Able to collect, track, and compare data Follow developmental milestones and evidence-based practice (eg, multiple linguistic hierarchy levels, and phonological processes)	Articulation Station: “It’s easy to use, has a plethora of target words to choose from, includes multiple linguistic hierarchy levels for students to practice their targeted sound at, and it even has a record and play back button for students to work on self monitoring and correcting.” Lively Letters-Phonics: “I'm a mom of two. One stronger reader and one weaker. They both LOVE this app! With letter sound stories, music, games and an opportunity to practice reading and spelling words...I will NEVER go back to flash cards again!” Speech Blubs: Language Therapy: “This app does not follow the typical developmental milestones of speech. It should start with early developing sounds to the early developing consonant-vowel combinations mixed with age appropriate play and functional communication words.” Word Vault Essential: “Would have been nice to know the app would erase all my students' data the day I decided not to continue the subscription. Trying to write IEPs and all profiles are gone.”
Receptive, expressive, and social language apps (Language Empires, Conversation Builder, ChatterPix Kids, Social Detective, Between the Lines Level 1 HD)	Integrate web-based data collection Ability to record and replay client answers Allow users to import individualized photos and voices Add reward systems (eg, trophies) as motivators Improve video and audio quality Reduce aversive sound effects Customize controls (eg, enable on or off) based on individual clients’ needs	Language Empires: “I use this app with my high school students. Before my students check their answers I have them explain their reasons for their choices. My students especially enjoy the vocabulary, why, inference and predicting sections. The data collection is perfect for IEP goal updates.” ConversationBuilder: “The different levels are helpful. Some students need the multiple choices of level one. Other students are ready for open-ended conversation turns. It is so interesting to compare the responses of my students with ASD to students who do not have difficulty with conversation.” ChatterPix Kids: “It helps my class because we are doing writing on animals and we are using the app to share...reports on our animals. It is also fun to play with friends on the app. But, one thing I’d change though would be to have more time to say what you want to say.” Social Detective: “The only criticism is that the sound effect for correct responses is aversive to some kids I know. They stopped playing the app because of the sound.” Between the Lines Level 1 HD: “One minor complaint I have is found within game mode. The child has no control over the aiming of the item used to throw and whilst this may remove frustration for some children, it causes great frustration for others. I would recommend a control feature to allow an “on or off” for this area based on the individual needs of the child.”

### Influential Factors for AAC and Speech-Language Apps

User feedback on both AAC and speech-language apps highlighted multiple influential factors shaping the perceptions and attitudes toward the apps. These factors include financial factors (eg, cost and pricing models), sociocultural factors (eg, multilingual capability and inclusive design in-app content), and ethical factors (eg, related to inclusive design). All AAC app users complained about the cost of the apps, except for the AAC app *CoughDrop*. In contrast to other AAC apps, which use a one-time app purchase of several hundred dollars, *CoughDrop* implemented a subscription-based model of US $6/month, which offered an alternative pricing model for AAC apps. This pricing model may have reduced the initial purchase cost burden for the users. AAC app users commented that, compared with other genres and categories of apps on the Apple App Store, the App Store lacks diversity on multiple devices despite the high cost. Users for some AAC apps (eg, *Speak For Yourself*, *LAMP WFL*, and *Tobii Dynavox Compass Connect*) have commented that these apps are priced too high given their limited customization or personalization capabilities (eg, *Speak for Yourself*). Other critiques include the argument that some apps demand too much time and storage to install (eg, *Tobii Dynavox Compass Connect*). With the change to a new Apple App Store design, more campaigns for specific apps have been featured, including apps for accessibility and health purposes. These likely made discounts more visible compared with before. For example, 1 user of *LAMP WFL* shared that “Last year iTunes offered this app half off on Autism Awareness Day.” App reviews for speech and language apps have relatively fewer complaints related to cost, which is likely owing to the different types of pricing models available. In contrast to AAC apps, which can cost nearly US $300, the most expensive speech-language app is less than US $50 for a one-time purchase. In addition, many apps offered free and pro versions. *Speech Blubs: Language Therapy* was the only app that used a US $9.99/month subscription-based model after a 7-day free trial. However, some users complained that their monthly subscription was charged for the whole year, whereas others commented that they were unable to afford the price during the COVID-19 pandemic. App users also reported mixed sentiment and contrasting perceptions regarding pricing for different speech-language apps (eg, *Word Vault Essential*, *Lively Letters-Phonics*). User perception of pricing is closely related to the amount and quality of app content, as users of apps such as *Social Detective* and *Articulation Station* both expressed their desire to have more content (eg, sound stimuli and video modules) for better replay ability and repeated use.

In addition to these financial factors, some user comments addressed factors related to cultural–linguistic diversity and ethical design for children. For example, reviewers for *Articulation Station* commented that the apps designed for younger learners should use age-appropriate words and eliminate certain images (eg, rifles and guns) to minimize exposure to violent content. One *LAMP WFL* user commented that “the Spanish vocabulary is a wonderful addition,” whereas another user of *Proloquo2Go* complained that:

Given the developers are from the Netherlands, I am surprised there is no Dutch language voice - only American, British and Indian English voices are available at present (although I would like to see more children's voices available in all these versions).

One reviewer for the speech-language app *Social Detective* stated that:

I work with primarily African-American students, so I find it troubling that the only person of color featured in the initial segment (16 video clips) of the app is an African-American tween male engaged in arguably the most overt “unexpected” behavior of all the children featured in the video clips within that segment. Perhaps I’m being too sensitive, but it strikes me as a subtle perpetuation of racial stereotypes.

Although such a report was only found in 1 review, it highlights the importance of inclusive design in app content for users from diverse cultural and ethnic backgrounds.

## Discussion

### Principal Findings

This study is one of the first to examine publicly available user reviews of speech-language therapy apps for children with communication disorders in the Apple App Store. Based on the analysis of a total of 418 reviews extracted across 16 apps written between 2009 and 2020, this study explored app content and features, as well as usability challenges related to both AAC apps and speech-language apps. Investigating user reviews regarding speech-language therapy apps informs app designers and developers who are interested in creating mobile apps for children with communication disorders that meet specific user needs and challenges. It also helps connect designers and developers with clinical recommendations based on SLPs’ evaluation of app qualities while working with children with communication disorders from a wide range of age groups and abilities. User reviews differ across the two genres of apps, with AAC apps being reported to have more issues with usability and speech therapy apps having more requests for additional app features to enhance clinical practice. Analysis of different stakeholder perspectives using AAC app reviews indicated that AAC apps gave users the ability to increase their communicative output. App reviewers praised AAC apps for giving their children a voice and the ability to interact with others, but also expressed that they would like to see improvements in app usability and accessibility. AAC app designers and developers should acknowledge features related to usability (eg, navigation and control) and appeal (eg, layout) and should focus on creating ease of learning, as well as programing and customization with compatibility across multiple hardware devices and systems (eg, mobile to desktop).

Reviews for speech-language apps also pointed to positive app content and features, as well as issues in usability for both children with communication disorders and their stakeholders. Primary complaints for speech-language apps were reported in areas of usability (eg, navigation and control and software and hardware compatibility) and appeal (eg, visual and audio features). Features such as child-friendly content and customization are highly preferred. Reviewers praised speech-language apps that followed evidence-based design practice guidelines and developmental milestones (eg, *Lively Letters-Phonics*), as well as apps that were available as supplementary companions to nondigital therapy materials (eg, *Social Detective*). Speech-language apps have dynamic visual features that make the apps engaging and entertaining for children to participate in speech therapy activities. As a result, these apps offered children pleasant experiences while completing speech activities at home as a carryover practice. In contrast, some apps (eg, *Speech Blubs: Language Therapy* and *Word Vault Essential*) were criticized owing to a lack of evidence-based design considerations in the app content. Therefore, it is highly recommended that speech-language app designers and developers consider collaborating with SLPs to implement developmentally appropriate app design practices [40] that are used during therapy sessions to align app features with evidence-based design.

The findings from this study contribute new insights regarding user experiences with different AAC apps and speech-language apps across multiple stakeholders (eg, parents, special education teachers, and clinicians). App Store reviews from different stakeholders further reinforced findings from prior ethnographic research that children with communication disorders interact with various assistive, educational, and even game apps as “a larger ecology of speech tools, including interactive games and apps” [13]. As many children with communication disorders are unable to communicate their needs directly owing to communication disorders, these reviews offer insight from and highlight the importance of adult stakeholders from the circle of care of children with communication disorders. These stakeholders not only share the use of apps, but also benefit from these apps as they support therapy intervention and home exercises outside the conventional therapy environments. Many recommendations in app reviews were specifically provided by parents and SLPs who thoughtfully explained their children and clients’ individualized needs across a range of communication areas. For parents, speech-language apps offer direct understanding regarding the therapy activities that their children can participate in to improve in different areas of communication (eg, articulation, language, and social pragmatics). Specific app features, such as data collection and visualization of progress across time frames (eg, day-to-day and month-to-month), provide parents with structured support to reinforce traditional therapy practices. Similarly, clinicians find value in tracking data within and across sessions, especially when working with clients who have different articulation and language goals. For SLPs, digitized speech-language apps also offer a greater portability across different settings compared with traditional paper-based therapy materials. As many apps can help increase engagement with therapy in children with communication disorders, clinicians are motivated to use speech apps as a dynamic way of teaching and targeting various goals. In addition, school-based SLPs have limited time with their clients and, therefore, often group clients with different types of disorders and levels of ability together. Apps that have the ability to collect data from clients (eg, *Language Empires*, *Between the Lines Level 1 HD*, and *Articulation Station*) and send data via emails for parents to view help bridge the home–school disconnect between different members of a child’s care circle. For app designers and developers, it is critical to evaluate these multi-stakeholder considerations in the app design and development process to support functional therapy activities across home, educational, and medical settings [41]. These user insights can be beneficial for app designers and developers to develop additional content and features that support clients who receive speech therapy better.

This study also compared multiple influential factors related to both AAC and speech-language apps. It was reported that many individuals who rely on AAC apps to communicate experience financial burdens when purchasing and maintaining the ongoing use of AAC apps, as nearly all AAC apps had user reviews related to the financial factors of app purchase and use. Speech-language therapy apps are typically offered as free or as paid pro versions, with only the *Speech Blubs: Language Therapy* not offering a free version (after a 7-day trial) and operating on a subscription-based pricing model. On the basis of these different pricing models across all apps, app designers and developers may need to attend to other marketing decisions across different revenue models, such as offering one-time purchases (eg, *Proloquo2Go* and *TouchChat HD*), subscription-based models (eg, *CoughDrop* and *Speech Blubs: Language Therapy*), or free versus paid versions. In addition, app reviews also revealed issues with a lack of representation of cultural and linguistic diversity in app content and the need for more consideration of inclusive design, an area that warrants additional research in the mHealth literature in general. Specifically, reviewers indicated that app content did not represent characters or linguistic variations from a variety of cultural and ethnic groups; as a result, individuals with linguistic variations and from minority groups can be marginalized by being unrepresented. It is important to note that only very few prior mHealth literature have considered the impact that cultural background can have on app user experiences. The latest research by Guzman et al [26] attempted to investigate cultural differences by looking for correlations between certain cultural backgrounds and various features of App Store reviews. Researchers have reported that sentiment, content, rating, and length differ at the country level and that these reviews follow specific cultural patterns. App designers and developers need to be sensitive to variations in cultural and linguistic patterns to design accessible and inclusive AAC and speech-language apps for children with communication disorders.

### Limitations

This study has several limitations that could be addressed in future research. First, although many apps have both iOS and Android versions, we only reviewed iOS apps owing to the high adoption of mobile devices (eg, the iPad) in the field of speech-language pathology. Second, as the goal of the study is to capture user insights from all app reviews over the period of the app’s history, the app analysis focused primarily on review content and did not specifically track or categorize app content and features mentioned in the review over different app versions. Third, we did not conduct any analysis with the star rating of each review, which may offer additional quantitative evaluation for app reviews. Fourth, many app reviewer identities remained unknown, as the reviewers did not disclose personal information (eg, whether they are an SLP or a parent) in their review. Therefore, we were unable to infer the backgrounds of all the people who wrote the reviews. Fifth, for speech-language apps, we only reviewed free versions to obtain more reviews; however, given that free versions are limited in the content offering compared with pro versions, this may contribute to the large distribution of reviews that include complaints about financial barriers and lack of comprehensive content. Sixth, this study only included clinician-recommended apps and likely neglected apps that are primarily used by caregivers and parents, who are also important stakeholders in their children’s speech and language development. Finally, despite our prior research indicating that clinicians use apps with their clients, clinicians’ app use does not necessarily indicate that these apps are empirically supported by research.

### Conclusions

Even with the vast number of iOS mobile apps for children with special needs, few research studies have investigated user insights regarding speech-language therapy apps designed for children with communication disorders. Owing to their communication disorders, the user experiences of children with communication disorders can be difficult to obtain and collect directly; however, analyses of App Store reviews from different stakeholders around the circle of care of children with communication disorders offers valuable information to researchers about specific app features that can support the communication development of children with communication disorders; the analyses also highlights usability issues that can be improved to reduce the frustration of children with communication disorders while using mobile apps. To our knowledge, this is the first study to analyze publicly available App Store reviews from different stakeholders (eg, SLPs, parents, and special educators) to examine pediatric speech-language therapy apps for children with communication disorders. These findings contribute to the understanding of desirable app content and features as well as the usability and accessibility issues with both AAC apps and speech-language apps. App reviews also revealed influential factors that highlight ongoing financial, sociocultural, and ethical and moral considerations for app design and development for children with communication disorders who need speech therapy. This study took place during the COVID-19 global pandemic, which resulted in lockdowns in many schools and clinics, preventing face-to-face therapy and resulted in more children with communication disorders learning from home with their caregivers. As many providers have transitioned to the use of remote learning and teletherapy, App Store reviews revealed that many of these speech therapy apps have supported different stakeholders, such as SLPs and parents, during remote learning and teletherapy. Future research should seek to develop in-depth analysis from these App Store reviews and evaluate individual app content and features to generate design insights that can best support communication through different types of service modalities, including teletherapy, in children with communication disorders.

## Appendix

Multimedia Appendix 1 Interviewees and mobile apps they use.

Multimedia Appendix 2 A list of 7 augmentative and alternative communication apps.

Multimedia Appendix 3 A list of 9 speech-language therapy apps.

Multimedia Appendix 4 Codebook for app store review.

## Abbreviations

AAC: augmentative and alternative communication
LAMP WFL: Language Acquisition Motor Planning Words for Life
SLP: speech-language pathologist
