# Supplementary material for: Listening to Stakeholders Involved in Speech-Language Therapy for Children With Communication Disorders: Content Analysis of Apple App Store Reviews
Source: JMIR Pediatr Parent. 2022 Jan 21;5(1):e28661. doi: 10.2196/28661 (PMC8817219; doi:10.2196/28661)
Supplement: Multimedia Appendix 2 [file pediatrics_v5i1e28661_app2.docx]

**Multimedia Appendix B: A List of 7 AAC Apps**

| **App Name & Developer** | **Price** | **App Description** |
| --- | --- | --- |
| [**CoughDrop**](https://apps.apple.com/us/app/coughdrop/id1021384570)  ([by CoughDrop, Inc](https://apps.apple.com/us/developer/coughdrop-inc/id1021376117))  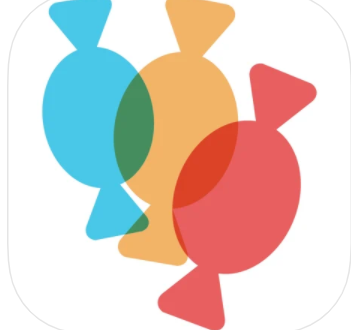 | Free (subscription: $6/month or $200 lifetime) | [CoughDrop](https://apps.apple.com/us/app/coughdrop/id1021384570) is an AAC app [by CoughDrop, Inc](https://apps.apple.com/us/developer/coughdrop-inc/id1021376117). The app was released in late November 2017 onto the iOS App store and is available for free purchase for users. CoughDrop is accessible across various devices including mobile phones, tablets, and computers. CoughDrop was designed specifically for individuals with CCNs and disorders including ASD, cerebral palsy, DS, Angelman syndrome, Rett syndrome. |
| [**GoTalk NOW**](https://itunes.apple.com/us/app/gotalk-now/id454176457?mt=8)  (by Attainment Company)  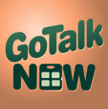 | $149.99 | [GoTalk NOW](https://itunes.apple.com/us/app/gotalk-now/id454176457?mt=8) is an AAC app released by [Attainment Company](https://www.attainmentcompany.com/info/about-attainment-company), a company founded in 1979 in Wisconsin. The company has a line of products which includes paper-based curricula, instructional videos, individualized education program (IEP) resources, and product lines for the aging population. It created their first assistive technology device (such as the “[Pocket Talker](https://www.attainmentcompany.com/personal-talker)”) and instructional software in 1993. |
| [**Language Acquisition through Motor Planning (LAMP) Words for Life**](https://itunes.apple.com/us/app/lamp-words-for-life/id551215116?mt=8) **(WFL)**  (by Prentke-Romich Company)  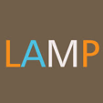 | $299.99 | [Language Acquisition through Motor Planning (LAMP) Words for Life](https://itunes.apple.com/us/app/lamp-words-for-life/id551215116?mt=8) (WFL) is an AAC app by [Prentke-Romich Company (PRC)](https://www.prentrom.com/our-history). It is the first app released by PRC, one of the most popular language acquisition AAC systems through evidence-based motor-planning principles. LAMP WFL can be used with a variety of age ranges and disorders such as Fragile X Syndrome and Apraxia of Speech (AOS). |
| [**Proloquo2Go**](https://itunes.apple.com/us/app/proloquo2go/id308368164?mt=8)  (by [AssistiveWare](https://www.assistiveware.com/products/proloquo2go))  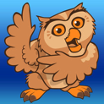 | $249.99 | [Proloquo2Go](https://itunes.apple.com/us/app/proloquo2go/id308368164?mt=8) is an AAC app developed by [AssistiveWare](https://www.assistiveware.com/), a company that was founded in 2000 in the Netherlands. AssistiveWare released the first full featured Augmentative and Alternative Communication (AAC) app for iOS in April 2009 on iPhone and iPod Touch. |
| [**Speak For Yourself**](https://apps.apple.com/us/app/speak-for-yourself/id482508198)  [(by Speak for Yourself LLC](https://apps.apple.com/us/developer/speak-for-yourself-llc/id482508201))  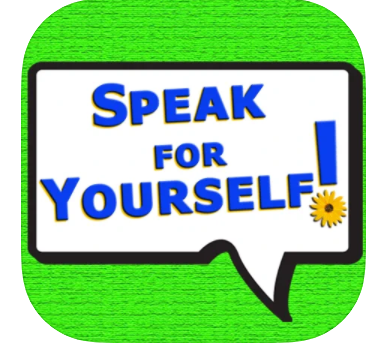 | $299.99 | [Speak For Yourself](https://apps.apple.com/us/app/speak-for-yourself/id482508198) is an AAC app [by Speak For Yourself LLC](https://apps.apple.com/us/developer/speak-for-yourself-llc/id482508201). The app was created and founded by two SLPs who specialize in AAC. The app became available on the app store in early November 2017 and utilizes evidenced-based practices (EBPs). Speak For Yourself is compatible with the iPad and iPhone. This app can be used with a variety of age ranges and disorders. |
| [**TouchChat**](https://apps.apple.com/us/app/touchchat-hd-aac/id398860728) **HD**  [(by Prentke Romich Company-Saltillo)](https://apps.apple.com/us/developer/prentke-romich-company/id474446992)  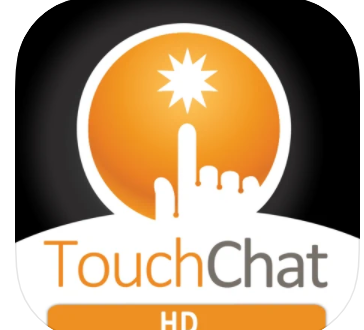 | $149.99 | [TouchChat](https://apps.apple.com/us/app/touchchat-hd-aac/id398860728) HD is an AAC app [by](https://apps.apple.com/us/developer/prentke-romich-company/id474446992) PRC-Saltillo. The app is designed for individuals with autism spectrum disorders (ASD), Down syndrome (DS), amyotrophic lateral sclerosis (ALS), AOS, and traumatic brain injuries (TBIs). The current version of the TouchChat HD app is available on iPhone, iPad, and iPod; it provides access to both English and Spanish languages within its inventory in both text and voices. |
| [**Tobii Dynavox Compass Connect**](https://apps.apple.com/us/app/tobii-dynavox-compass-connect/id692773734)  [(by Tobii Dynavox LLC)](https://apps.apple.com/us/developer/tobii-dynavox-llc/id446210474)  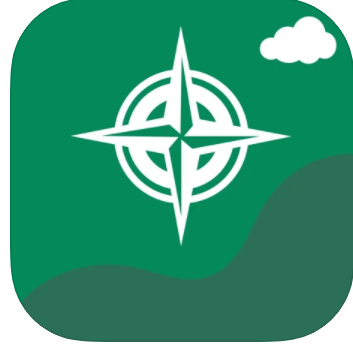 | $179.99 | [Tobii Dynavox Compass Connect](https://apps.apple.com/us/app/tobii-dynavox-compass-connect/id692773734) is an AAC app [by Tobii Dynavox LLC](https://apps.apple.com/us/developer/tobii-dynavox-llc/id446210474). The app was released late August 2013 by one of the leaders within assistive technology for both communication and eye tracking. Tobii Dynavox Compass Connect is compatible with the iPad, iPad Mini, Windows Tablet and offers a free trial on the iOS App store. It is designed using evidenced-based research and tailored for users of all ages. The app can be used with populations with ASD, cerebral palsy, aphasia, AOS, DS, developmental disabilities, ALS and neurological disorders, TBI, and those with CCNs. |
