# Supplementary material for: Listening to Stakeholders Involved in Speech-Language Therapy for Children With Communication Disorders: Content Analysis of Apple App Store Reviews
Source: JMIR Pediatr Parent. 2022 Jan 21;5(1):e28661. doi: 10.2196/28661 (PMC8817219; doi:10.2196/28661)
Supplement: Multimedia Appendix 3 [file pediatrics_v5i1e28661_app3.docx]

**Multimedia Appendix C: A List of 9 Speech and Language Apps**

| **App Name & Developer** | **Price** | **App Description** |
| --- | --- | --- |
| [**Articulation Station**](https://itunes.apple.com/us/app/articulation-station/id467415882?mt=8)  (by Little Bee Speech)  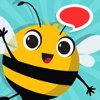 | Free  (Pro version: $59.99) | Articulation Station is an app developed by Little Bee Speech which offers clinicians to work on articulation skills. It is a comprehensive articulation app that offers practice at the word, sentence and story levels. The free version offers content for speech sound /p/; the Pro version offers the complete English speech sound inventory. |
| [**Between the Lines Level 1**](https://itunes.apple.com/us/app/between-the-lines-level-1-hd/id574405272) **HD**  (by Hamaguchi Apps)  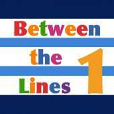 | $24.99 | Between the Lines Level 1 HD is an app developed by Hamaguchi Apps. The app offers clinicians to work on pragmatic language skills through the use of photos, audio clips and short video clips. |
| [**ChatterPix Kids**](https://itunes.apple.com/us/app/chatterpix-kids/id734046126?mt=8)  [(by Duck Duck Moose)](https://itunes.apple.com/us/app/chatterpix-kids/id734046126?mt=8)  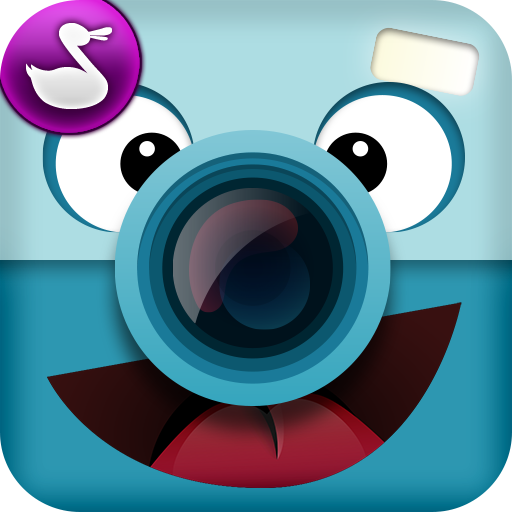 | Free | ChatterPix Kids is an app developed by Duck Duck Moose. This app targets social language in constructing conversation through story retells that are customizable with individualized pictures and voices. |
| **ConversationBuilder**  (by Mobile Education Store LLC)  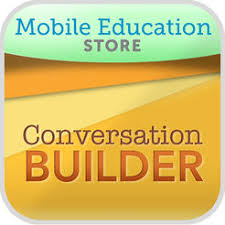 | $19.99 | ConversationBuilder is an app developed by Mobile Education Store LLC. This app targets social skills that include social interactions, social stories, and conversation skills with general speech and language elements such as vocabulary development, sentence production, and social awareness. |
| [**Language Empires**](https://itunes.apple.com/us/app/language-empires/id562910097?mt=8)  (by Smarty Ears, LLC)  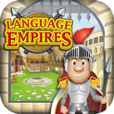 | $29.99 | Language Empires is an app developed by Smarty Ears, which allows clinicians to work on multiple language skills using a medieval city theme. |
| **Lively Letters - Phonics**  (by Telian-Cas Learning Concepts Inc.)  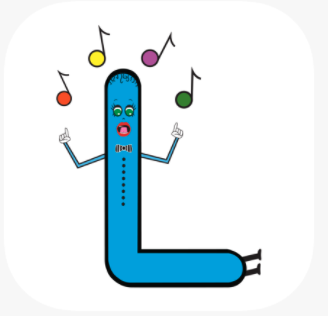 | $19.99 | Lively Letters - Phonics is an app developed by Telian-Cas Learning Concepts Inc. This app focuses on the development of phonics and phonological awareness for individuals as early as 2 years of age. |
| [**Social Detective**](https://apps.apple.com/us/app/social-detective/id975189305)  [(by Social Skills Builder Inc.)](https://apps.apple.com/us/app/social-detective/id975189305)  [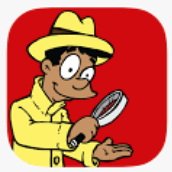](https://apps.apple.com/us/app/social-detective/id975189305) | $9.99 | Social Detective is an app developed by Social Skill Builder, Inc. This app targets social language and behaviors for children with social-communication disabilities. |
| **Speech Blubs: Language Therapy**  (by Blub Blub Inc)  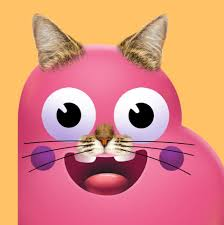 | Free  (subscription: $9.99/month) | Speech Blubs: Language Therapy is an app developed by Blub Blub Inc. It is a voice-controlled speech therapy app designed to help your child learn new sounds and words, and to practice speaking in a stimulating, educational environment. The app offers a 7-day free trial and a $9.99/month subscription charge. |
| [**World Vault Essential**](https://apps.apple.com/us/app/word-vault-essential/id947222381)  [(by](https://apps.apple.com/us/app/word-vault-essential/id947222381) HomeSpeechHome PLLC)  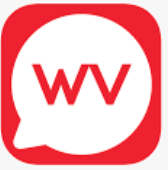 | Free  (Pro version: $49.99) | Word Vault Essential is an app developed by HomeSpeechHome PLLC. This app targets general language elements that include articulation, social vocabulary, phonology, and pragmatics. |
