# Supplementary material for: Listening to Stakeholders Involved in Speech-Language Therapy for Children With Communication Disorders: Content Analysis of Apple App Store Reviews
Source: JMIR Pediatr Parent. 2022 Jan 21;5(1):e28661. doi: 10.2196/28661 (PMC8817219; doi:10.2196/28661)
Supplement: Multimedia Appendix 4 [file pediatrics_v5i1e28661_app4.docx]

**Multimedia Appendix D: Codebook for App Store Review**

| Codes  (2nd level) | Sub-Codes  (1st level) | Explanation | [App Name, Date for Review] Example Reviews |
| --- | --- | --- | --- |
| Client Characteristics: qualities that describe clinicians, clients, or other stakeholders who were mentioned their user experience related to the apps | Age or Grade Group | Client’s age or grade level | - [Proloquo2Go, 2012-05-18] My four year old is perfectly happy to use the iPhone version (however she has good eyesight, hearing and fine-motor skills). - [Lively Letters - Phonics, 2017-07-15] I can't wait to use this app with my early elementary school students! |
|  | Type of Disability | Users’ disorders or disabilities reported by reviewers | - [Language Empires, 2016-08-13] Mixed language groups - [TouchChat HD 2019-10-17] I have CP. - [Speech Blubs: Language Therapy, 1/28/20] Diagnosed with a speech delay |
|  | Levels of Ability | Descriptions related to the severity of (e.g., mild, moderate, severe) | - [Articulation Station, 2017-09-04] Minimal language - [ConversationBuilder 2011-04-10] I have used it with a few students with ASD along with students with language impairments. I even find it useful for my students with articulation impairments - [LAMP WFL, 2014-01-16] she is nonverbal and communicates using sign language. While she still uses sign language, the range of her communication is expanding |
|  | Length of Use | Client’s length of use with the app | - [Word Vault Essential 2015-05-21] I use this daily and can’t live without it! |
| Clinician Characteristics: information related to providing services to CwCD using mobile apps | Clinical Setting | Schools, private practice, etc. | - [Between the Lines Level 1 HD, 2013-10-25] SLP in private practice |
|  | Clinician Location | Physical location of the clinician | - [Lively Letters - Phonics, 2017-11-10] I began my career using Lively Letters in CT. I have since moved to Houston, TX |
|  | Clinician’s Speciality | Clinician’s area of expertise | - [Lively Letters - Phonics, 2020-07-17] I’m a reading specialist and dyslexia interventionist |
|  | Length of Use | Clinician’s length of use with the app | - [Articulation Station, 2020-04-07] I have been using this app for the last five years. |
| Clinical Practice | Intervention area and domain | Intervention areas and domains that is being targeted through app use | - [Language Empires, 2019-04-30] I like that this app addresses lots of language skills - [Between the Lines Level 1 HD, 2013-05-03] to assist students struggling with social cues and nuances |
|  | Therapy goals and activities | Specific objectives and activities that apps are being targeted | - [Word Vault Essential, 2015-05-12] I use this app a TON with students to develop social vocabulary and stimulate various social scenes - [Word Vault Essential, 2018-02-15] This app is great for many things including articulation, language, phonology, and pragmatics. |
|  | Workplace productivity | Factors related to workplace productivity (e.g., the individual's time deliverance in programming the device or in communicating) | - [Social Detective, 11-29-2016] I love the social detective curriculum so I downloaded the app, but I am very disappointed. My client answered all of the questions in about 10 minutes |
|  | Evidence-based practice and research | Factors related to speech therapy techniques/research that can be proven effective through evidence-based practice | - [Speech Blubs: Language Therapy, 6/21/17] it does not seem to align with the first words kids typically begin to say during language development - [Speech Blubs: Language Therapy, 6/21/17] this app was well thought out to align with speech therapy and does not seem to be in alignment with research. - [Speech Blubs: Language Therapy, 11/10/19] does not follow developmental speech milestones |
| Additional Stakeholders | Technical personnel | People involved with the design and development of apps such as developers, customer service of patrons | - [Articulation Station, 2020-04-28] emailed the developer. Super-fast response. Chris provided top-notch support |
|  | Non-technical personnel | People not directly involved with the app design or customer service team (e.g., parents, teachers, and other professionals) | - [Articulation Station, 2020-04-28] this lovey little app is only available to my daughter if daddy is home |
| Influential Factors | Financial | Factors related to individuals’ finances with affordance of apps | - [Articulation Station, 2020-03-01] The free version only gives you the letter P all other numbers cost $3.99 a piece or the full version for $59 |
|  | Sociocultural | Factors related to individuals’ sociocultural interests or needs with the affordance of apps | - [Proloquo2Go, 2019-04-02] Purchased to learn Spanish |
|  | Ethical and moral | Factors related to individuals’ wants and needs | - [Proloquo2Go, 2020-02-04] Has the ability to be backed up if device is destroyed, can just get a new ipad and quickly replace a child’s/adults voice. We all have the right to speak and this App provides not only accessibility but also can be personalized to the individual and skill level. |
| App Characteristics | App Genre | Category in which apps fall under such as the names of apps | - [TouchChat HD, 2016-05-10] TouchChat is an awful app. Proloquo2go and Speak for Yourself |
|  | App Use Technique | How the SLP, teachers, and/or family members uses an app | - [Articulation Station, 2016-09-22] our team utilize this APP to supplement skilled activities and enhance sessions. - [Lively Letters - Phonics, 2020-07-17] Track their progress. |
|  | App Content | The information presented and displayed in the app (e.g., speech sounds, types of vocabulary) | - [Articulation Station, 2020-07-02] I absolutely love how they include all speech sounds and can be used in either initial medial or final position as well as at the word, phrase, sentence and story level! |
|  | Data Management | The collection, curation/storage, representation, and exporting/reporting of user data | - [Language Empires, 2016-08-13] you can record all of your data on-line while playing the game! - [Between the Lines Level 1 HD, 2013-11-14] Data is recorded for each student per session. Results can printed or emailed. |
| [Usability Issues](https://docs.google.com/presentation/d/1SMN_-QyI2KzZyggaOVmxUq6-E_ar3CfUOHqFX_AJIIM/edit#slide=id.p)  Refers to issues with the degree in which a user is able to learn, control, and understand an app | Control | the degree in which a system allows a user to manage and have an appropriate level of sensitivity and responsiveness that are related to the proficiency and experience of the individual using an app | - [Language Empires, 2019-04-10] Group those with different language levels - [Language Empires, 2019-05-21] Change difficulty for clients by setting the number of answer choices |
|  | Errors | Problems with usability which may lead to frustrating experiences for users and may lead to a negative effect on the overall quality and success of an app | - [Proloquo2Go, 2019-10-20] the sensitivity on this app is extremely annoying. - [Language Empires, 2014-12-20] Visual distraction and hard to focus - [Lively Letters - Phonics, 2019-01-28] Will not play the flash cards |
|  | Aesthetics | Sound quality, visual designs, the overall look and appeal | - [Articulation Station, 2019-10-02] The stimulus items are visually interesting - [Proloquo2Go, 2018-11-19] Adding own photos - Sound quality for the unvoiced phonemes is improved and it runs with greater speed and ease [2017-11-10] |
|  | Customization | The ability to allow users to configure an entire interface or select pages to meet their needs for use | - [TouchChat HD, 2016-05-10] editing is really cumbersome - [Lively Letters - Phonics, 2011-09-06] Editing buttons affects other screens |
|  | Accessibility  (more prevalent in AAC apps) | The ability to allow information and interface functions be accessed and used by people with disabilities | - [Lively Letters - Phonics, 2017-05-10] Portable - [Tobii Dynavox Compass Connect, 2013-10-25] They are able to communicate faster; While it takes some time to set up it is worth it |
| Device Issues | Hardware (compatibility, disk memory, etc.) | Hardware component, a physical characteristic, or a physical part of the technical device | - [TouchChat HD, 2013-12-24] We use this app on our daughters iPad, it works pretty well. I wanted to add the app to my phone for a more mobile option. - [Tobii Dynavox Compass Connect, 2018-05-20] I downloaded it on my new iPad and when I try to open it, the wheel just spins and then it closes down |
|  | Software (version, cloud storage) | The programmed application and its functionality and capabilities | - [Articulation Station, 2020-04-28] I upgraded to Pro on my iphone - [Word Vault Essential, 2015-05-21] Can’t wait to get the PRO version with pictures |
| Recommendation | App Referral | Information about app referrals and purchase information | - [Lively Letters - Phonics, 2020-07-17] recommended it to my friend who has a 3 year old - [Articulation Station, 2020-07-02] I was first introduced to this app by a few Speech-Language Pathologists during there therapy session. |
|  | Suggestions for Improvement | Ideas to make existing issues better | - [Between the Lines Level 1 HD, 2013-11-12] Fix bad language - [LAMP WFL, 2013-04-17 ] Options for speech rate and voice personalization would be useful so the user can learn to speak clearly and slowly. |
|  | Feature Request | Additional features suggested by reviewers in allowing for a better user experience and effective intervention | - [Lively Letters - Phonics, 2020-07-20 ] I wish there were a way to pause the story or song and then start playing again in the same place you left off - [TouchChat HD, 2019-10-17] can you please add iPadOS voices! |
